# Pathologically Proven Papillary Fibroelastomas Attached to the Left Atrial Appendage and Pulmonary Vein Ridge

**DOI:** 10.1016/j.jaccas.2024.103192

**Published:** 2025-03-05

**Authors:** Omar W. Aboukhatwa, Elias Akiki, Hayat Memis, Kathryn F. Larson, Eugene L. Scharf, Lawrence J. Sinak, Vuyisile T. Nkomo, Melanie Bois, Joseph Maleszewski, Kyle Klarich, Reto Kurmann

**Affiliations:** a Department of Cardiovascular Medicine, Mayo Clinic, Rochester, Minnesota, USA; b Department of Neurology, Mayo Clinic, Rochester, Minnesota, USA; c Department of Laboratory Medicine and Pathology, Mayo Clinic, Rochester, Minnesota, USA

**Keywords:** cardiac tumors, coumadin ridge, echocardiography, left atrial appendage, papillary fibroelastoma

## Abstract

Papillary fibroelastomas (PFEs) are the most common benign cardiac tumors, typically found on valvular surfaces but less frequently on extravalvular structures. Despite their benign nature, PFEs are associated with serious embolic events. This study analyzes 13 cases of pathology-proven PFEs in the left atrial appendage (LAA) and pulmonary vein ridge. Data on patient demographics, clinical presentations, imaging, and histopathology were reviewed. The median patient age was 73 years, with slight male predominance. Tumor sizes ranged from 2 mm to 20 mm. Twelve PFEs were detected via transesophageal echocardiography, and neurologic events were observed in 4 patients. This case series emphasizes the importance of maintaining a broad differential diagnosis for LAA masses because PFEs can easily be mistaken for thrombi.

## Visual Summary

**Figure:**
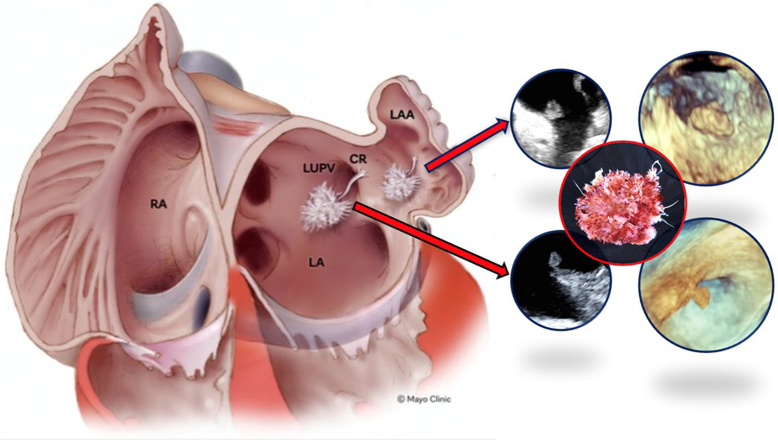

Papillary fibroelastomas (PFEs) have recently been identified as the most common benign cardiac tumors, often found on valvular surfaces, most commonly the aortic valve (65%) followed by the mitral valve (13%).^1^ However, PFEs can also attach to extravalvular structures, including the left atrial appendage (LAA) and its surroundings. These neoplasms are best detected using transesophageal echocardiogram (TEE). Despite their benign nature, they have been associated with a range of serious embolic events.

**Learning Objectives**

• To consider PFE in the differential diagnosis of LAA masses due to its embolic potential
• To understand that accurate identification is crucial to guide appropriate treatment with usually surgical removal to prevent serious complications

Advances in imaging techniques have led to the increased recognition of intracardiac masses attached to atypical sites. The LAA is a well-known site for thrombus formation, often associated with embolic strokes. A distinct anatomical structure, known as the pulmonary vein ridge (PVR), also referred to as the coumadin ridge or Q-tip, has recently garnered attention in the literature.

The PVR is a fibrous ridge located in the left atrium, specifically between the LAA and the left superior pulmonary vein. Due to its shape and position, it is frequently mistaken itself for a tumor or thrombus, which explains its association with the anticoagulant medication coumadin, commonly used to mitigate stroke risk.

Tumors in the LAA and the PVR pose significant diagnostic challenges due to the complex anatomy of the region. Misidentification can result in unnecessary, costly treatments for patients. Thus, accurate identification and classification of masses in this area are critical for appropriate management, yet remain clinically challenging. Herein, we present 13 cases of PFEs attached to the LAA and PVR.

## Case 1

**Figure 1:**
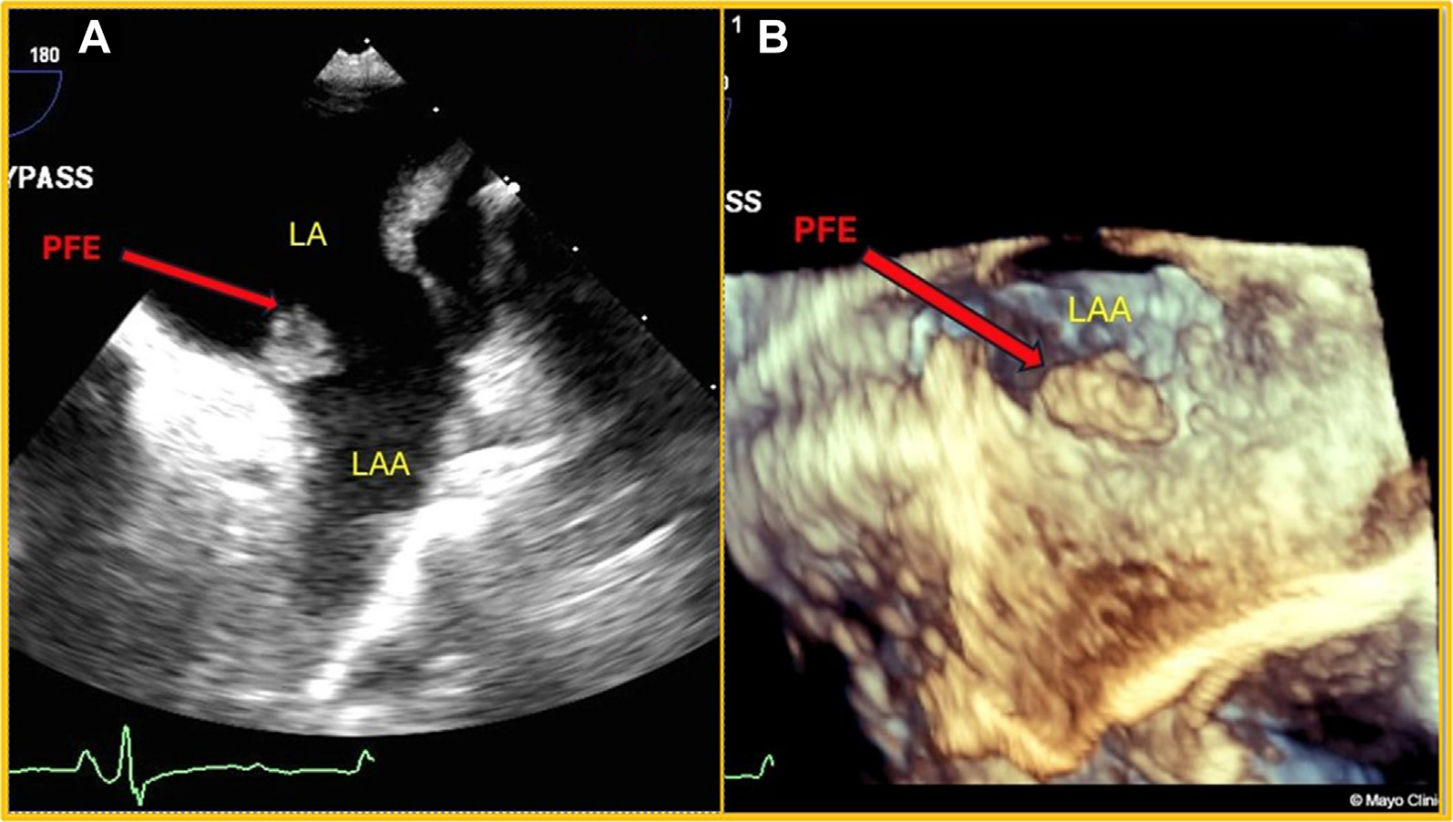
Transesophageal Echo (A) TEE, 2-D TEE midesophageal view of LA with focus on LAA: PFE attached to the orifice of LAA at the junction of the mitral annulus. (B) TEE, 3-D mode-focus on LAA: en-face view of PFE attached to the orifice of LAA. 2-D = 2-dimensional; 3-D = 3-dimensional; LA = left atrium; LAA = left atrial appendage; PFE = papillary fibroelastomas; TEE = transesophageal echocardiography.

A 69-year-old woman with a history of 2 strokes presented with presyncopal and syncopal spells that had gradually increased in frequency. She was admitted for a TEE assessment to evaluate a potential embolic source. An 8- × 7-mm mass was detected attached to the orifice of the LAA (Figures 1A and 1B, Video 1). The mass was initially suspected to be an atrial myxoma. She underwent surgery for mass excision and LAA occlusion, along with coronary artery bypass graft surgery (CABG) for significant coronary artery disease (CAD). Histopathologic examination confirmed the mass to be a PFE. The patient recovered well postsurgery. Follow-up echocardiograms showed no remaining mass.

**Visual Summary:**
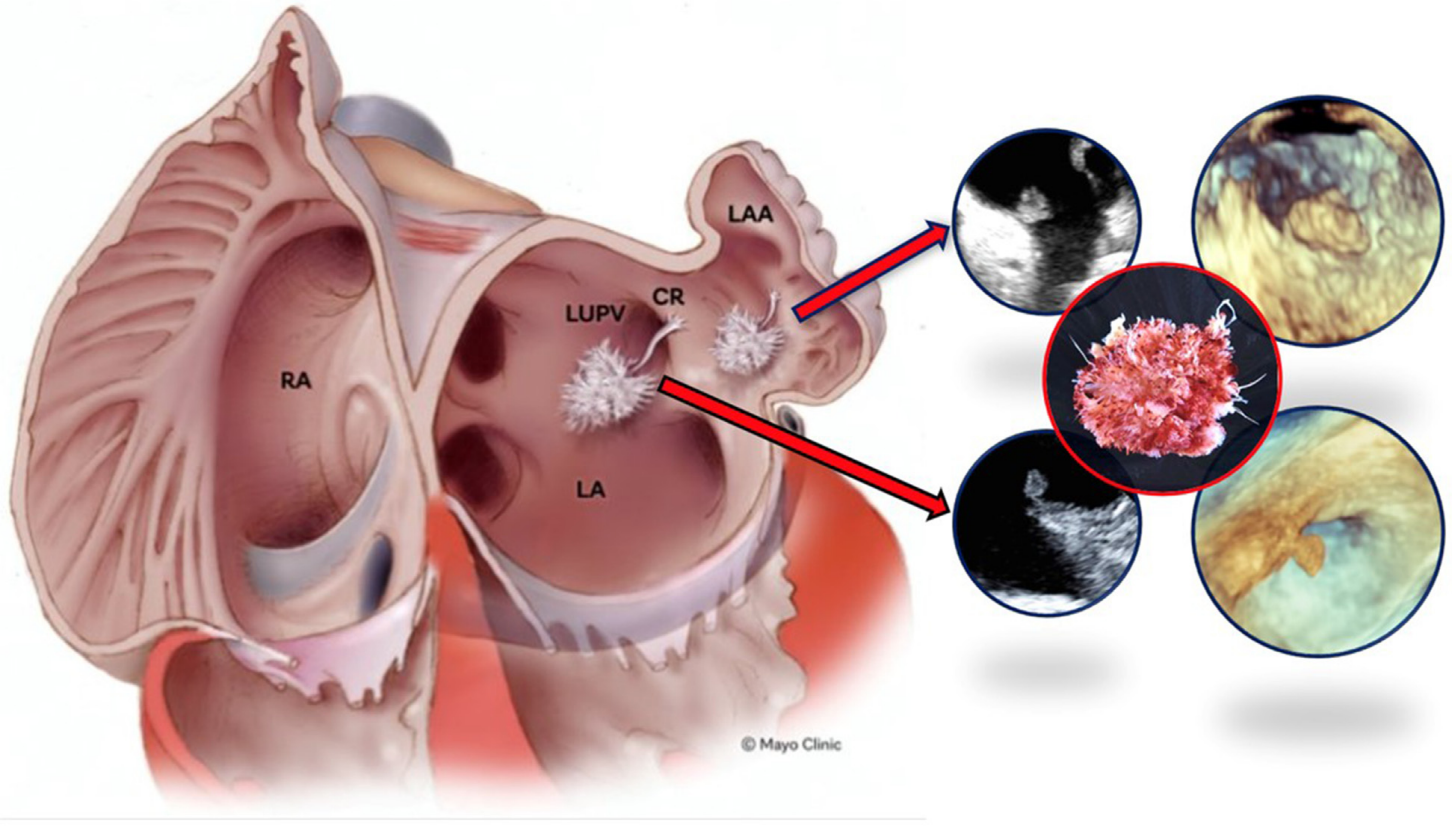
The Locations of the PFEs and the Anatomy of the LAA and CR in Relation to the LUPV, LA, and RA CR = Coumadin ridge; LA = left atrium; LUPV = left upper pulmonary vein; PFE = papillary fibroelastoma; RA = right atrium.

## Case 2

A 74-year-old man with a history of unprovoked pulmonary embolism, an embolic stroke diagnosed on brain magnetic resonance imaging (MRI), and atrial fibrillation (AF) on therapeutic anticoagulation, presented for mitral valve repair due to severe regurgitation post–robotic mitral valve repair. During the intraoperative repair of the mitral valve, the surgeon noted a mass in the LAA. The intraoperative TEE did not detect the mass due to its small size (2 mm). The mass, along with the LAA, was excised and, on pathologic examination, a small PFE was diagnosed. The patient experienced 3 episodes of AF with rapid ventricular response, all successfully managed with amiodarone infusion. A transient elevation in creatinine levels was observed but quickly returned to baseline. The patient was discharged 10 days postsurgery.

## Case 3

A 68-year-old man with a history of episodes of transient ischemic attack (TIA) and AF had undergone multiple direct current cardioversions, all of which failed to maintain sinus rhythm. He was evaluated as an inpatient and recommended to undergo a maze procedure. During the presurgical examination, a tiny 1- × 3-mm mass was identified in LAA attached by a stalk on 1 of the TEEs. Therefore, he underwent a robotically assisted removal of the mass in addition to the maze procedure, and the mass was confirmed to be a PFE. Postoperatively, he developed AF with rapid ventricular response. Despite treatment with amiodarone and beta-blockers, his rhythm could not be stabilized, necessitating electrical cardioversion to restore normal sinus rhythm. He made a good recovery, and follow-up TEE revealed no residual mass.

## Case 4

**Figure 2:**
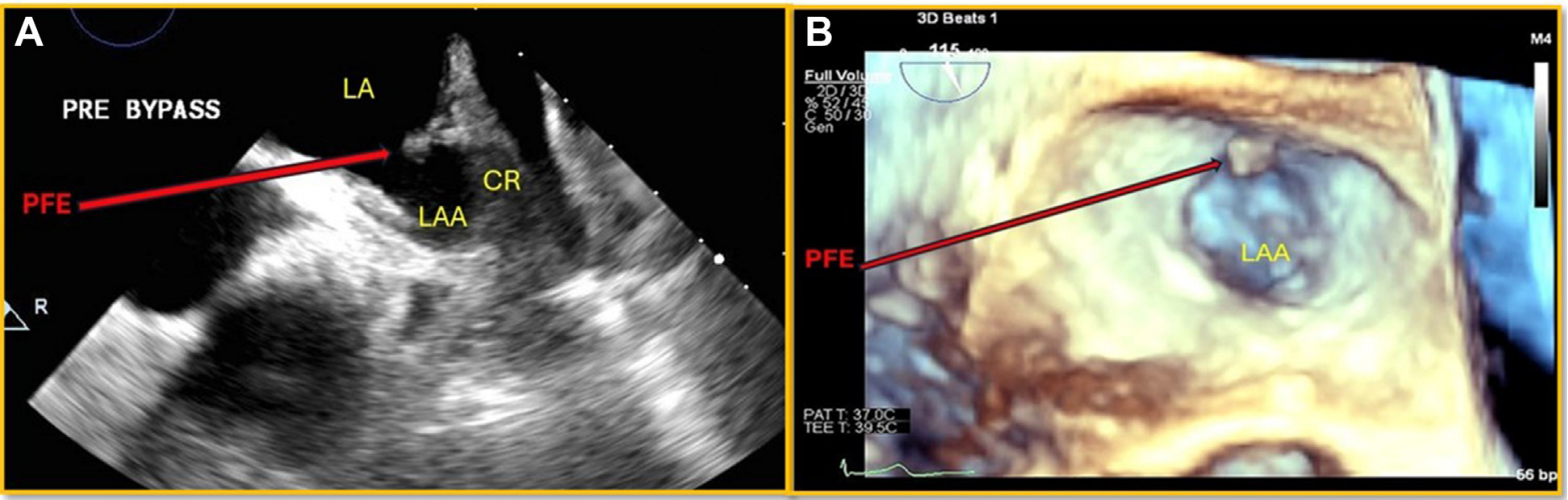
Transesophageal Echo (A) Intraoperative 2-D TEE: globular echodensity attached to the PVR. (B) 3-D TEE, en-face view of LAA orifice: mobile mass attached to the PVR. CR = coumadin ridge; PVR = pulmonary vein ridge; other abbreviations as in Figure 1.

A 73-year-old man presented with anginal chest pain. He had a past medical history notable for an episode of TIA. Computed tomography (CT) and MRI of the brain were unremarkable. As part of the investigation of a cardiac source of an emboli, a TEE was performed, revealing a 7- × 4-mm mobile mass near the base of the PVR (Figures 2A and 2B, Video 2). Therapeutic anticoagulation was immediately initiated. A follow-up TEE was done 3 months later, which showed no change in the mass. The mass was, therefore, surgically excised and confirmed to be a PFE. During his postoperative course, he experienced intermittent junctional rhythms, sinus bradycardia, as well as episodes of AF and complete heart block. Consequently, a temporary transvenous pacemaker was inserted. After this, a permanent pacemaker was implanted to manage tachy-brady syndrome. He was discharged 1 week later.

## Case 5

A 73-year-old woman had a medical history of valvular heart disease, atrial flutter on long-term therapeutic anticoagulation, hypertension, and hypothyroidism. She was hospitalized for a retropubic abscess, which was treated with surgical drainage and intravenous antibiotics; blood cultures at the time were negative. During hospitalization, a TEE was done to exclude infective endocarditis, which incidentally revealed a mass attached to the PVR. Subsequent serial TEEs were performed while maintaining therapeutic anticoagulation and antibiotic treatment, revealing that the mass remained stable in size during 8 months. To prevent embolic events and determine the nature of the mass, surgical excision of the mass and the LAA was performed. Pathologic examination confirmed the mass to be a PFE. The follow-up TEE revealed no residual mass, and the patient made an uneventful recovery.

## Case 6

**Figure 3:**
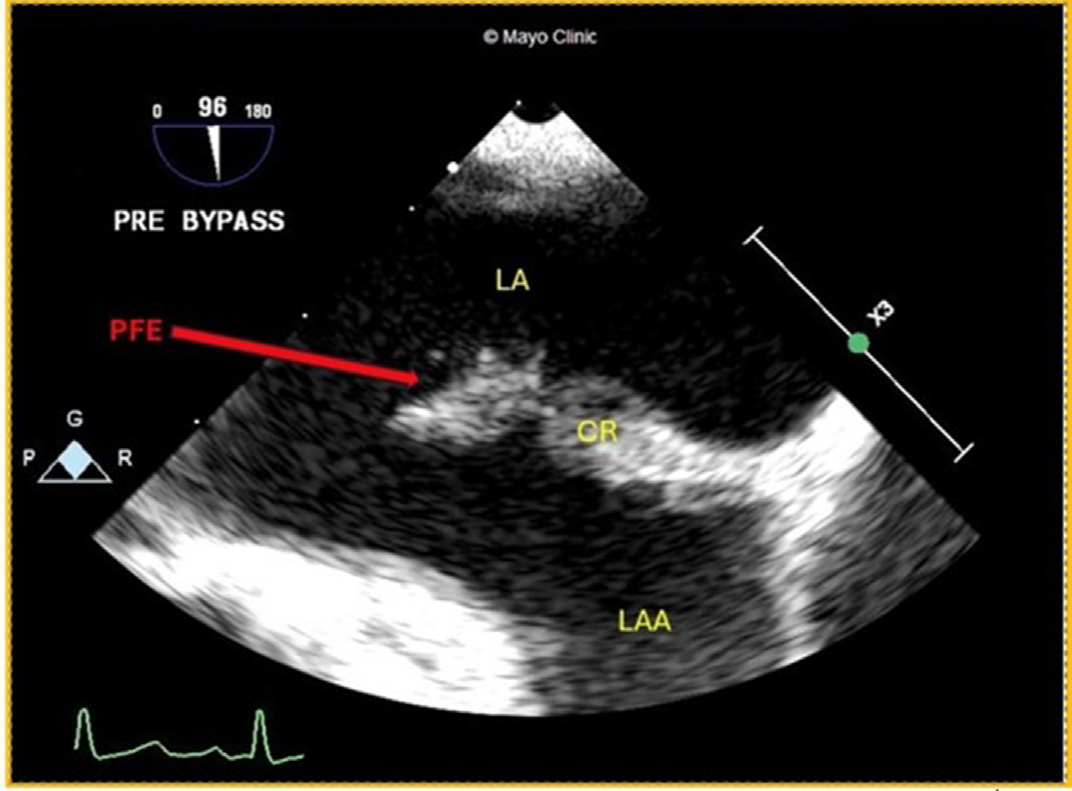
Transesophageal Echo TEE, 2-D mode-focus on LAA, showing the PFE attached to the PVR by a stalk. Abbreviations as in Figures 1 and 2.

A 78-year-old woman with moderate aortic stenosis and CAD presented with progressive dyspnea on exertion and fatigue for the past year. TEE revealed a 14- × 10-mm mobile mass with frond-like projections attached by a stalk to the PVR of the LAA, suspicious for a PFE (Figure 3, Video 3), alongside a calcified mitral annulus with moderate aortic valve stenosis. She underwent aortic valve replacement (AVR), CABG, and removal of the LAA mass, which confirmed the diagnosis of a PFE. Postoperative TEE detected no residual mass. The patient experienced 1 episode of AF after surgery, which was successfully terminated with an amiodarone infusion. Therapeutic anticoagulation was initiated, and the patient made an uneventful recovery.

## Case 7

A 41-year-old woman presented with exertional dyspnea and dizziness upon standing. Her cardiac history includes chronic AF, complete atrioventricular canal defect, secundum atrial septal defect, and patent ductus arteriosus, all of which were completely repaired during her childhood.

**Figure 4:**
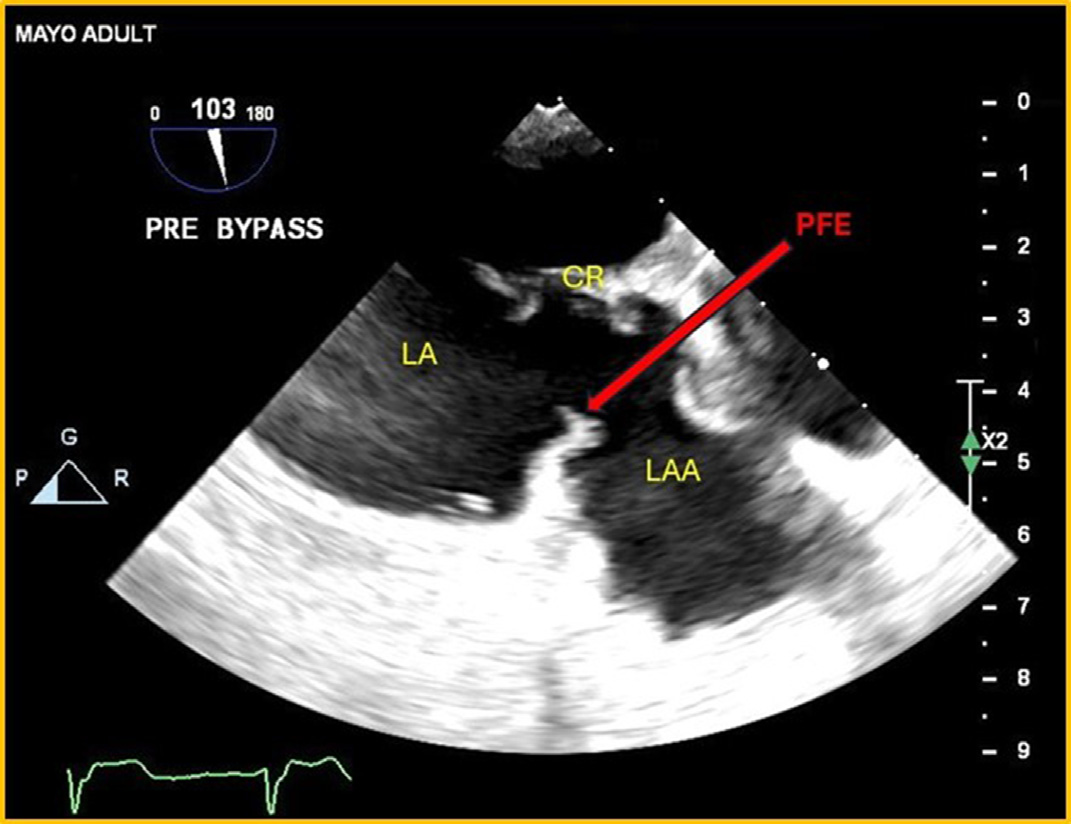
Transesophageal Echo Intraoperative TEE, 2-D mode-focus on LAA and coumadin ridge, dilated LA with a large mobile filamentous mass attached to the orifice of LAA. Abbreviations as in Figures 1 and 2.

An echocardiogram revealed severe mitral valve regurgitation, a severely enlarged left atrium, and a left ventricular ejection fraction of 51%. The patient was scheduled for mitral valve repair. During the intraoperative TEE, a mobile filamentous mass was visualized within the very large LAA, initially thought to be a thrombus (Figure 4, Video 4). The mobile portions were resected and sent for pathologic examination, where a PFE was confirmed. Her postoperative course was uneventful, and she made an uneventful recovery.

## Case 8

**Figure 5:**
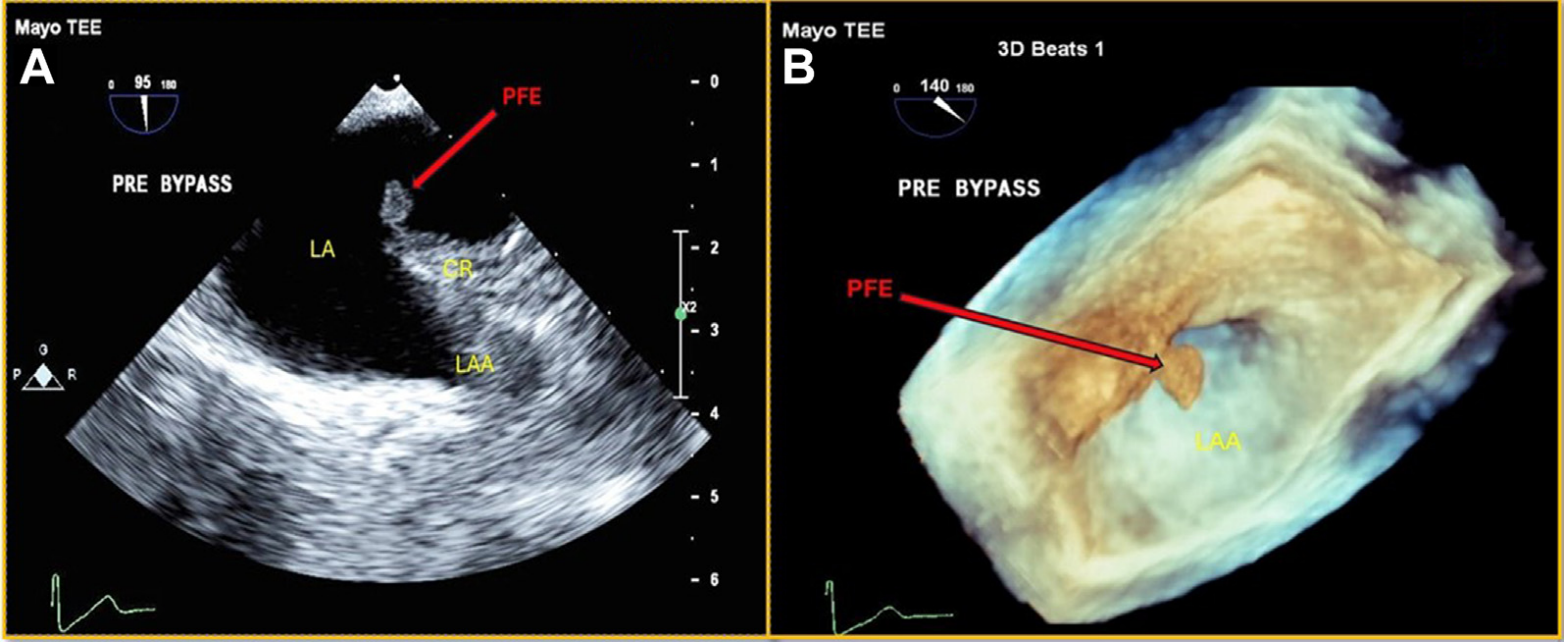
Transesophageal Echo (A) TEE, 2-D mode-focus on LAA, showing a PFE on the coumadin ridge at the apex of the reflection of LAA and LUPV tissue. (B) 3-D transesophageal echo of a PFE attached to the orifice of the LAA en face. LUPV = left upper pulmonary vein; other abbreviations as in Figures 1 and 2.

An 87-year-old woman with a history of histoplasmosis and Graves’ disease treated with radioiodine presented with progressive exertional dyspnea. She was diagnosed with progressive aortic stenosis, and basal septal hypertrophy was noted. She underwent an uncomplicated tissue AVR and septal myectomy. A small 4- × 6-mm left atrial mass attached to the PVR was found on intraoperative TEE (Figures 5A and 5B, Videos 5 and 6), which was also resected. On examination of the tissue, a PFE was confirmed. Postoperatively, the patient developed a transient left bundle branch block and AF, both of which spontaneously resolved. A follow-up TEE revealed no residual mass.

## Case 9

**Figure 6:**
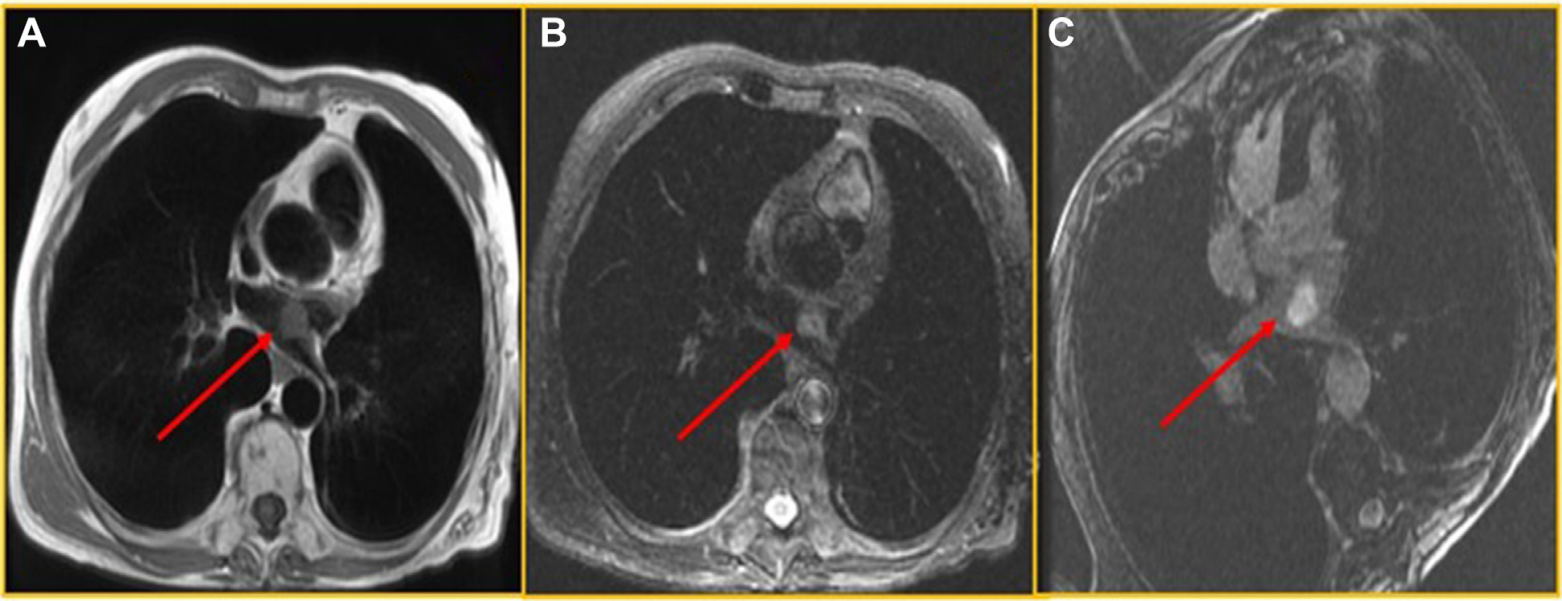
Cardiac Magnetic Resonance (A) T1-weighted image showing iso-intense mass in the LA lateral wall. (B) T2-weighted image showing mildly hyperintense at the lateral wall of the LA. (C) Delayed myocardium enhancement imaging shows avidly enhancing mass in the LA. Abbreviations as in Figure 1.

**Figure 7:**
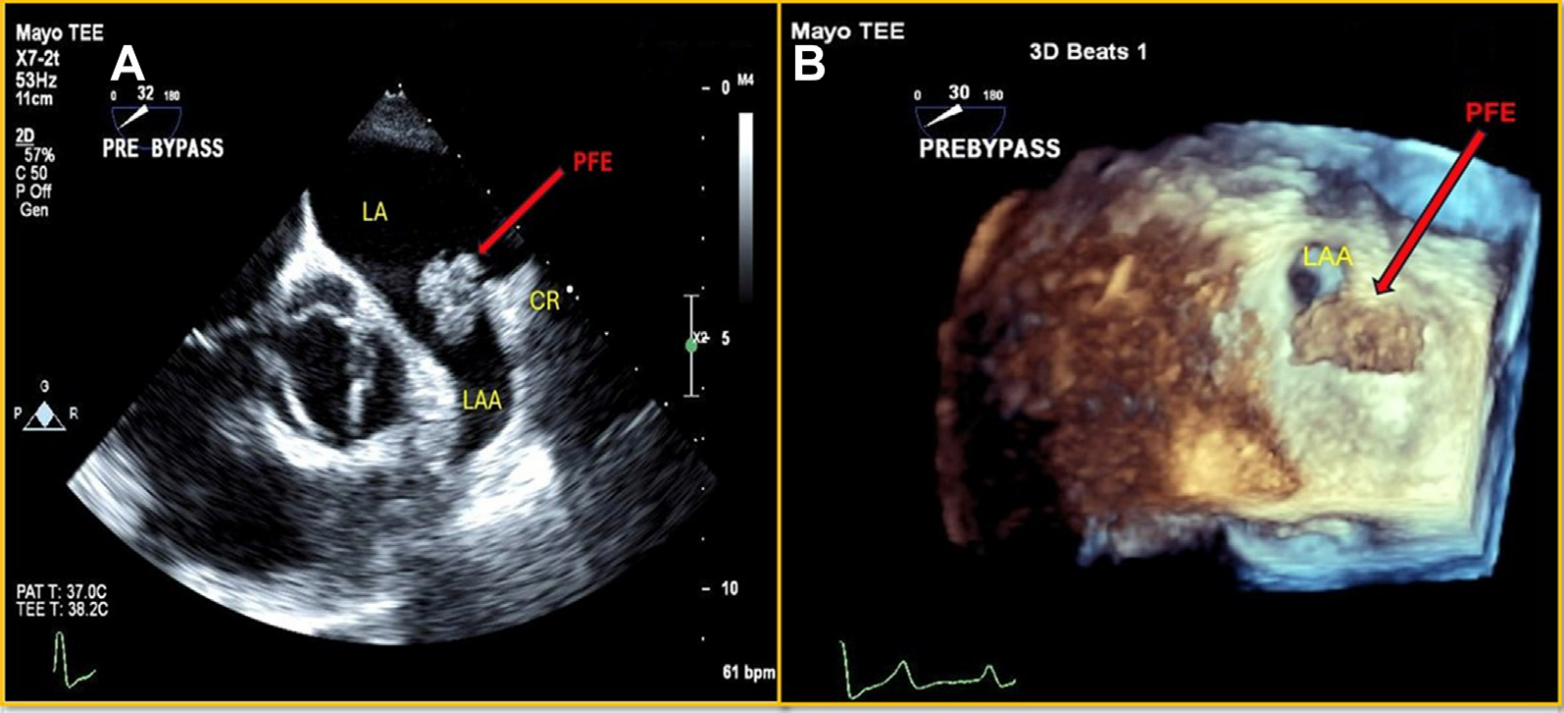
Transesophageal Echo (A) Intraoperative 2-D TEE, focus on LAA showing a mass attached to PVR. (B) Intraoperative 3-D TEE, focus on LAA showing a mass attached to PVR. Abbreviations as in Figures 1 and 2.

A 75-year-old man diagnosed with adenocarcinoma of the gastroesophageal junction had undergone neoadjuvant radio-chemotherapy. During the initial evaluation for the adenocarcinoma, a cardiac MRI incidentally revealed a 20- × 17-mm mass in the left atrium (Figure 6). The imaging suggested the mass could be a myxoma or fibroma. A positron emission tomography scan showed no increased metabolic activity within the heart, indicating the mass was not positron emission tomography–avid. The patient’s medical history included a nephrectomy for renal cell carcinoma more than a decade earlier, raising concerns that the atrial mass might be metastatic from the renal cell carcinoma. A TEE confirmed the presence of the mass at the PVR, with thrombus being an additional differential (Figures 7A and 7B, Video 7). The patient was subsequently started on therapeutic anticoagulation. Despite treatment, the mass remained stable in size during a 7-month follow-up period. Surgical resection was eventually performed, and histopathologic analysis identified the mass as a PFE. He made a quick recovery with no postoperative complications.

## Case 10

**Figure 8:**
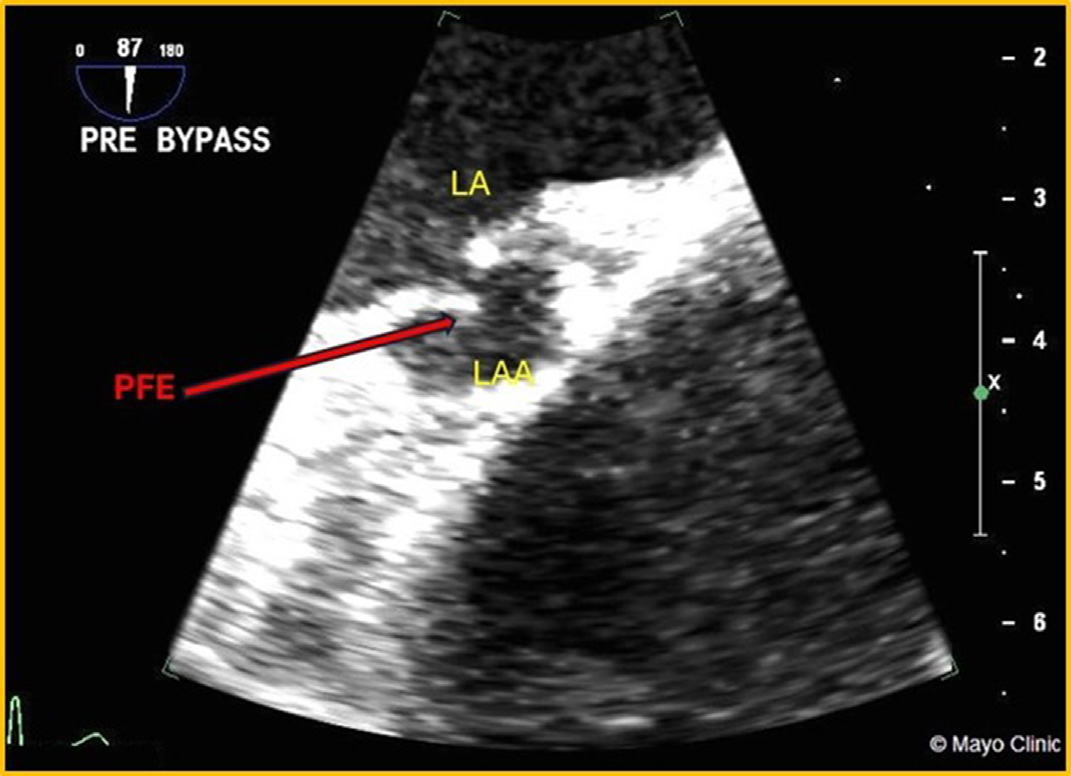
Transesophageal Echo (Left Atrial Appendage Mass-Zoomed) Intraoperative TEE revealed an incidental small mobile fibrinous strand attached to the orifice of the LAA. Abbreviations as in Figure 1.

A 65-year-old woman with a history of limited scleroderma with CREST syndrome was being evaluated for AVR in the setting of progressive aortic stenosis. During surgery, intraoperative TEE revealed an incidental 5-mm mobile fibrinous strand attached to the orifice of the LAA (Figure 8). The mass was excised and subsequently confirmed to be a PFE. Postoperatively, no mass was detected on TEE, and she made a full recovery with no postoperative complications.

## Case 11

**Figure 9:**
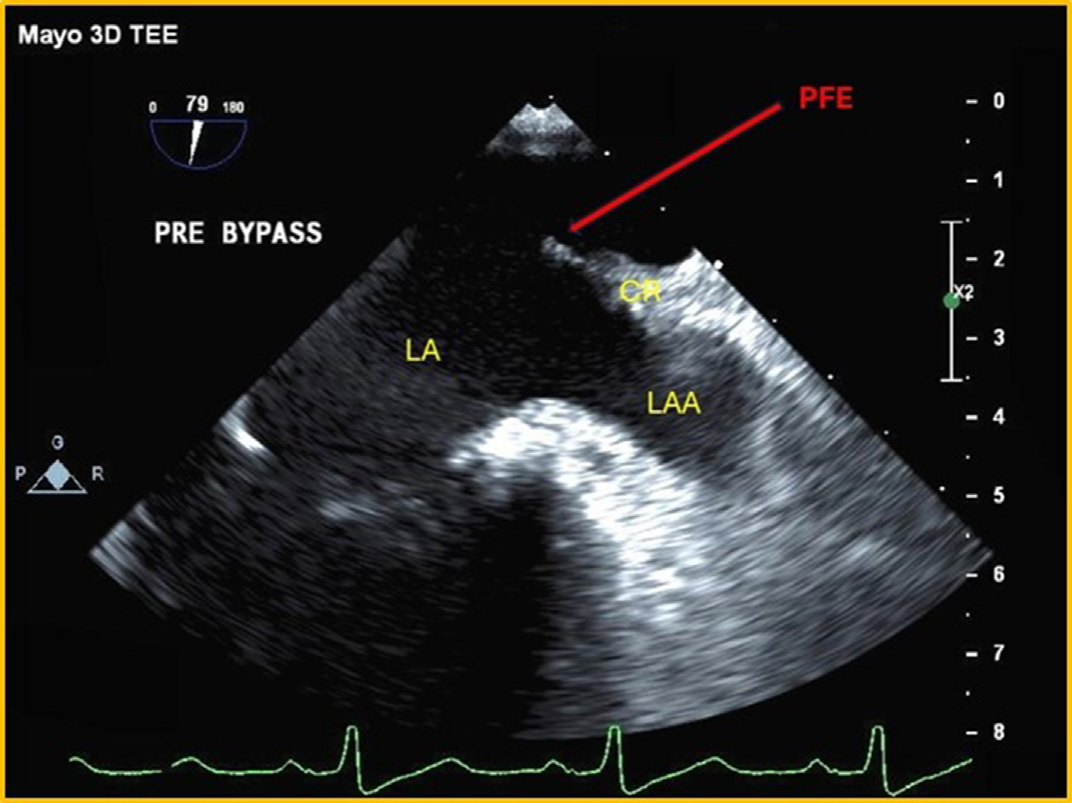
Transesophageal Echo Intraoperative 2-D TEE, focus on LAA: mass attached to the PVR. Abbreviations as in Figures 1 and 2.

A 79-year-old man presented with a 10-day history of exertional dyspnea. His medical history was significant for recently diagnosed congestive heart failure (ejection fraction: 31%) and diffuse CAD. A coronary angiogram showed extensive multivessel disease not amenable to stenting, and, therefore, the patient was evaluated for potential CABG. During a 2-vessel CABG and septal myomectomy, a LAA mass attached to the Q-tip was incidentally noted on intraoperative TEE and excised, along with ligation of the LAA (Figure 9, Video 8). The mass was initially thought to be a thrombus but was proven to be a PFE on histopathology. Follow-up echocardiograms revealed no residual mass, and the patient had an uneventful recovery.

## Case 12

**Figure 10:**
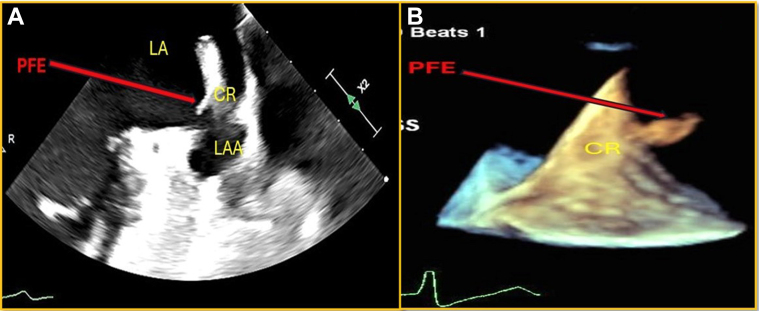
Transesophageal Echo (A) Intraoperative 2-D TEE: globular echodensity attached to the PVR. (B) Intraoperative 3-D TEE, focus on LAA mass attached to the PVR. Abbreviations as in Figures 1 and 2.

A 73-year-old man with hypertrophic obstructive cardiomyopathy, presented with exertional dyspnea. A TTE showed dynamic left ventricular outflow tract obstruction with resting gradient of 64 mm Hg, with an increase to 100 mm Hg with Valsalva. Despite medical therapy, he continued to experience dyspnea and, therefore, was scheduled for surgical septal myomectomy. No preoperative TEE was performed. During intraoperative TEE, a 6- × 4-mm mobile, shimmering, globular echodensity was seen attached to the PVR at the LAA (Figures 10A and 10B). It was excised and was later confirmed to be a PFE by pathologists. He was discharged 1 week later after an uneventful recovery.

## Case 13

**Figure 11:**
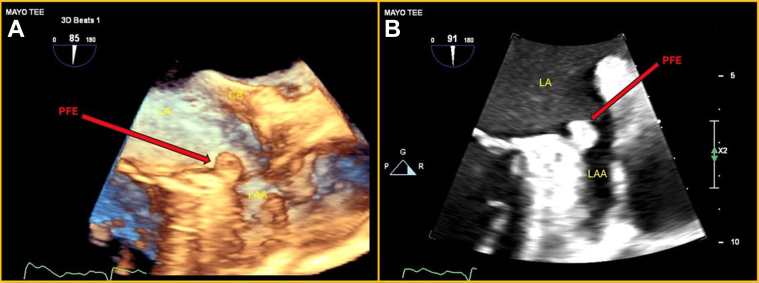
Transesophageal Echo (A) Intraoperative 2-D TEE: globular echodensity attached to the PVR. (B) 3-D TEE, en-face view of LAA orifice: mobile mass attached to the coumadin ridge. Abbreviations as in Figures 1 and 2.

A 70-year-old man presented with acute exacerbation of congestive heart failure with reduced ejection fraction from CAD. His medical history was notable for mixed pre–post–capillary pulmonary hypertension, permanent AF on warfarin, chronic kidney disease, and metabolic syndrome. A TEE revealed severe mitral valve regurgitation due to restriction of the posterior leaflet and mitral annular dilation. An echo-dense mobile mass (12 mm × 8 mm) was attached to a stalk protruding from the LAA, suggesting a possible PFE or myxoma (Figures 11A and 11B, Video 9). Blood cultures showed no growth. The patient subsequently underwent mitral valve replacement, CABG, and LAA mass excision. Histopathologic examination confirmed the PFE diagnosis. However, the hospital course was complicated by challenging acute decompensated heart failure and persistent hypoxic respiratory failure secondary to pneumonia. Despite receiving guideline-directed medical therapy, the patient’s condition deteriorated, ultimately leading to a cardio-respiratory arrest that was unresponsive to resuscitation efforts.

## Discussion

This case series highlights 13 patients with pathologically proven PFEs attached to the LAA, with 7 cases attached specifically to the PVR. The median age of the cohort was 73 years, with a slight male predominance (54%). The tumor sizes ranged from 2 mm to 20 mm, with a median size of 5 mm. This variability in size highlights the challenge in diagnosing these masses, particularly when attached to less common sites like the LAA and PVR, also known as the coumadin ridge or Q-tip of the left atrium.

PFEs are traditionally considered rare, but the increasing detection rate of these masses underscores the importance of advanced imaging techniques such as TEE and cardiac MRI. In our study, TEE was pivotal in the initial visualization of 12 of 13 of the PFEs. Only 1 case was diagnosed only postoperatively by pathologists after the excision of the LAA, illustrating the complementary and indispensable role of pathology to confirm the diagnosis.

### Clinical Presentation and Risk Factors

Although most patients with PFE are asymptomatic, in our cohort, the majority experienced dyspnea, likely attributable to concurrent cardiac comorbidities. Four patients presented with neurologic events (stroke/TIA), with 2 having a prior history of stroke. One patient exhibited symptoms of both TIA and angina pectoris, suggesting PFEs as a potential source of embolism. Only 1 patient reported chest pain or palpitations, whereas the remaining 4 patients were asymptomatic. The symptomatic presentation in these patients highlights the clinical significance of PFEs, given their potential to cause embolic events.

The prevalence of cardiovascular comorbidities and atherosclerotic risk factors in our cohort was notable: 69% had hyperlipidemia, 38% had AF with 53% of the cohort being therapeutically anticoagulated for at least 3 months presurgically, 31% had CAD, 54% had hypertension, and 46% were former smokers. The demographic, clinical characteristics, and imaging modalities used to assess these patients are summarized in Table 1.

**Table 1:** Clinical and Imaging Characteristics of Patients With Papillary Fibroelastomas

Case	Age	Sex	Mass Size	Neurologic Event				Clinical Presentation					Diagnostic Modality				
				CVA	TIA	AF	Anticoagulated (>3 mo Presurgically)	Chest Pain	Syncope/Presyncope	Dyspnea	Palpitations	Asymptomatic/Follow-Up on Other Condition	Routine TEE (Cardiac Evaluation)	Intraoperative TEE	CMR	Found Only on Surgical Inspection	Other Cardiac Surgery Performed
1	69	Female	8 × 7mm	Yes	Yes	No	No	No	Yes	No	No	No	Yes				CABG
2	77	Male	2 × 1mm	Yes	Yes	Yes	Yes^b^	No	No	Yes	Yes	No				Yes	MV repair
3	60	Male	1 × 4mm	No	Yes	Yes	Yes^b^	No	No	No	No	Yes	Yes				X
4	73	Male	7 × 3mm	No	Yes	No	Yes^a^	Yes	No	No	No	No	Yes				X
5	73	Female	5 × 5mm	No	No	Yes	Yes^b^	No	No	No	No	Yes	Yes				X
6	78	Female	14 × 10mm	No	No	No	No	No	No	Yes	No	No	Yes				CABG + AVR
7	41	Female	1 × 4mm	No	No	Yes	Yes^b^	No	Yes	Yes	No	No		Yes			MV replacement + TV repair
8	87	Female	4 × 6mm	No	No	No	No	No	No	Yes	No	No		Yes			AVR + septal myectomy
9	75	Male	20 × 17mm	No	No	No	Yes^a^	No	No	No	No	Yes			Yes		X
10	65	Female	5mm	No	No	No	No	No	No	No	No	Yes		Yes			AVR
11	79	Male	9 × 4mm	No	No	No	No	No	No	Yes	No	No		Yes			CABG
12	73	Male	6 × 4mm	No	No	No	No	No	No	Yes	No	No		Yes			Septal myectomy
13	70	Male	12 × 8 mm	No	No	Yes	Yes^b^	No	No	Yes	No	No	Yes				MV replacement
Sum				2	4	5	7	1	2	7	1	4	6	5	1	1	

AF = atrial fibrillation; AVR = aortic valve replacement; CABG = coronary artery bypass grafting; CMR = cardiac magnetic resonance; CVA = cerebrovascular accident; MV = mitral valve; SOE = standard of care echocardiography; TEE = transesophageal echocardiography; TIA = transient ischemic attack; TV = tricuspid valve.

a Patient was anticoagulated presurgically due to the mass discovered.

b Patient was anticoagulated presurgically due to AF.

### Embolic Risk

**Figure 12:**
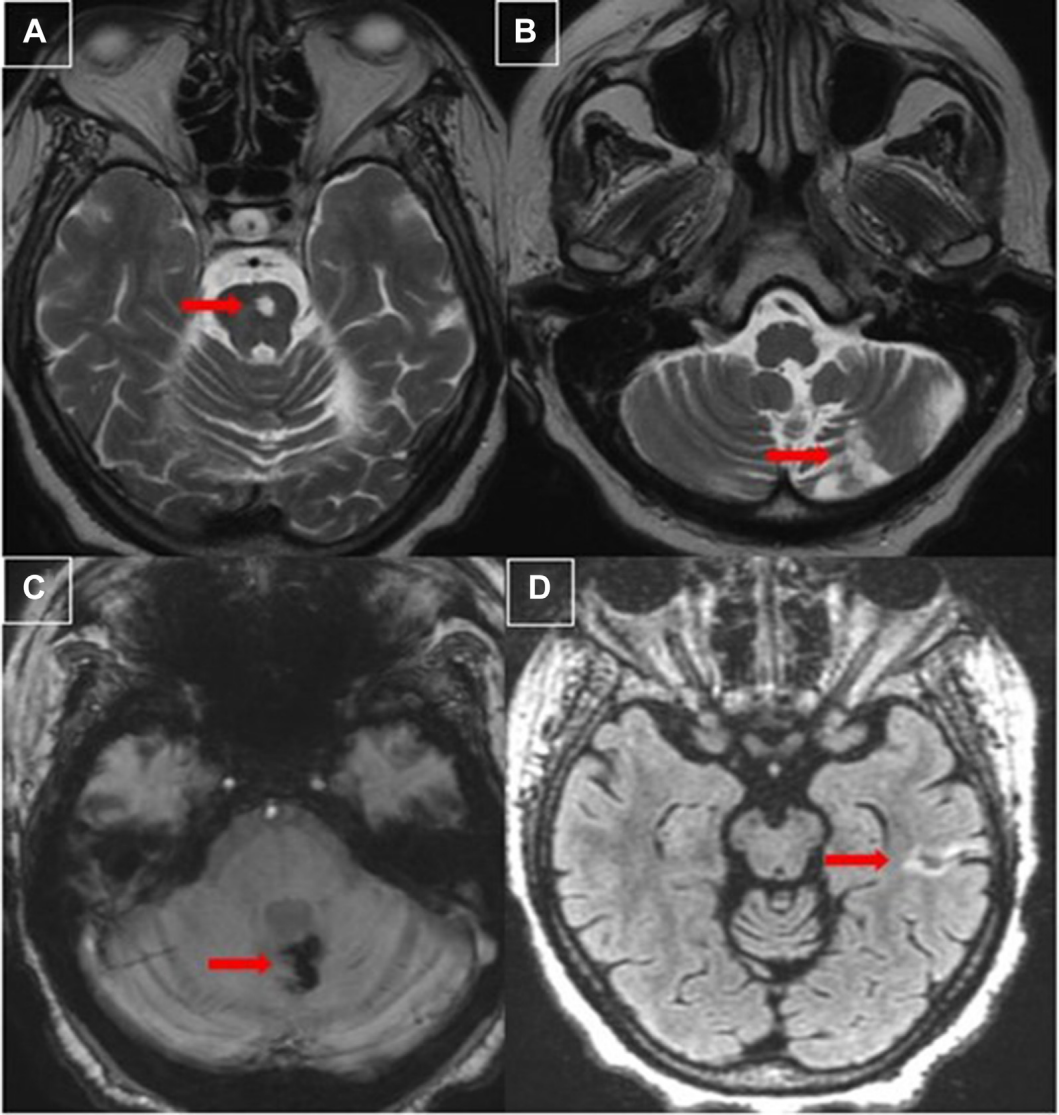
Head MRI (A) Axial T2 weighted sequencing, chronic left pontine infarction with cavitation (red arrow). (B) Axial T2 weighted sequencing suggests chronic infarction of the left cerebellar hemisphere consistent with prior embolism (red arrow). (C) Susceptibility weighted imaging axial reconstruction demonstrates foci of hemosiderin deposition in the left cerebellar hemisphere and vermis consistent with hemorrhagic transformation of a prior ischemic infarct (red arrow). (D) Axial T2 weighted Fluid Attenuated Inversion Recovery (T2 FLAIR) demonstrates gliosis of the left inferior temporal lobe consistent with chronic infarction (red arrow). MRI = magnetic resonance imaging.

In this case series, 4 patients experienced neurologic events—2 presented with TIAs and 2 with overt strokes (Figure 12)—underscoring the embolic potential of PFEs. Despite their benign nature, the mobile, frond-like morphology of PFEs, particularly those attached by stalks, may increase the risk of embolization, as demonstrated in these cases alongside symptoms of angina. Notably, 3 of the 4 stroke cases involved PFEs attached to the LAA orifice. It has been suggested that the shape and mobility of PFEs might be linked to an increased risk of embolism.^2^ However, the precise factors that predispose certain PFEs to embolization remain unclear. Currently, no conclusive evidence ties specific morphologic features, such as size, mobility, or attachment site, to an elevated embolic risk. Ongoing studies aim to clarify these factors and establish guidelines for evaluating embolic risk in patients with PFEs.

### Diagnostic Challenges

A significant proportion of the cases (7 of 13) were incidentally discovered intraoperatively using TEE, and 4 were visualized on TEE performed for various reasons, including valvular assessment, assessment of intracardiac source of embolism, exclusion of infective endocarditis, and CAD assessment. One case was incidentally detected on cardiac MRI performed as part of a metastatic tumor work-up, whereas 1 case was too small to be detected by any imaging modality and was only diagnosed postoperatively by histopathology. These findings confirm that PFEs can often be discovered accidentally, posing a diagnostic and therapeutic challenge.

The LAA is a common site for thrombus formation, particularly in patients with AF. In cases of persistent LAA thrombosis observed in multiple follow-up TEEs despite therapeutic anticoagulation, it is crucial to consider PFE as a differential diagnosis. Persistent thrombosis in the LAA can mimic the appearance of other cardiac masses, complicating diagnosis and potentially delaying appropriate treatment. Distinguishing between these conditions is essential because the management strategies differ significantly.

Complementary imaging techniques, such as cardiac MRI and CT, can be invaluable in these situations. These modalities provide detailed anatomical information and tissue characterization that can help differentiate between thrombi and other masses. Cardiac MRI, for instance, can offer insights into tissue composition and vascularity, potentially identifying features that distinguish a PFE from a thrombus or myxoma.^3^ It can also be used to evaluate the characteristics of cardiac tumors by visualizing their relationship with surrounding tissues, playing a crucial role in surgical planning, assessing tumor progression, and monitoring for postoperative recurrence.

CT imaging can also aid in this differentiation by providing high-resolution images that clarify the mass’s location and attachment site. These imaging modalities, used alongside TEE, enhance diagnostic accuracy and guide clinical decision-making.

Incorporating these advanced imaging techniques into the diagnostic workflow can lead to earlier identification of PFEs and facilitate the appropriate therapeutic interventions. This approach emphasizes the importance of a thorough and nuanced evaluation in cases where LAA thrombosis does not resolve with standard treatment, ultimately improving patient outcomes.

### Pathology

**Figure 13:**
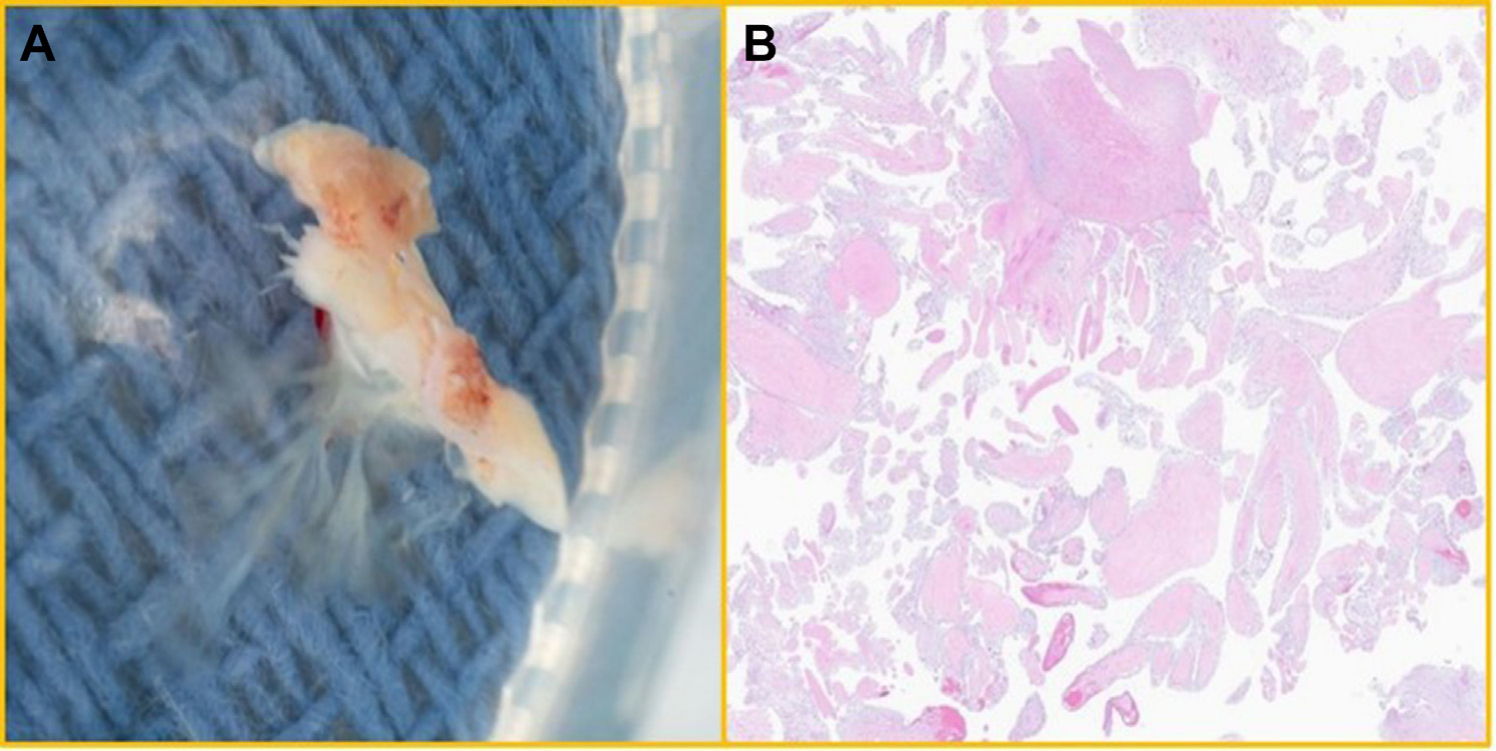
Pathology (A) Gross pathology of resected PFE from the PVR showing papillary fronds with sea anemone–like appearance. (B) Histopathology of resected PFE showing thin branching papillary fronds composed of collagen fibrosis and elastic tissue, surrounding by a layer of endothelium. Abbreviations as in Figures 1 and 2.

Histopathologic analysis remains the definitive method for diagnosing a PFE. Gross examination typically reveals small, pedunculated masses (2 mm up to 5 cm) with a distinctive sea anemone–like appearance, characterized by delicate papillary fronds (Figure 13A). Microscopically, when examined using hematoxylin and eosin staining, these fronds consist of avascular, collagen-rich connective tissue with a central fibroelastic core, which may contain collagen, elastic fibers, and occasional mucinous elements.^4^ The papillary projections are lined by a layer of endothelium (Figure 13B). Although no malignant features are observed, some molecular studies have identified KRAS mutations, suggesting a possible oncogenic component in some PFEs.^5^

### Implications and Future Directions

The increased detection rate of PFEs in uncommon locations highlights the necessity of a multidisciplinary approach in the managing these patients. Collaboration among cardiologists, cardiac surgeons, radiologists, and pathologists is essential to ensure accurate diagnosis and optimal treatment strategies.

Advanced imaging techniques play a crucial role in the early detection and characterization of PFEs, facilitating timely intervention. In cases where valvular masses are suspected to be PFEs, our group advocates for blood cultures and thrombophilia testing to exclude nonbacterial thrombotic endocarditis and infective endocarditis.^4^^,^^6^^,^^7^ The utility for this testing in masses on the PVR remains less clear. Monitoring the growth of these masses may offer insights, although PFEs typically exhibit slow growth.^8^

Myxomas have also been noted in the LAA (unpublished, Mayo Clinic), further complicating the differential diagnosis of masses in this region. Potential differential diagnoses for LAA masses include PFE, myxoma, thrombus, endocardial fibroelastosis, vegetations, and left atrial pseudotumor.^9^

Accurate diagnosis of PFEs is vital to prevent misidentification and unnecessary treatments, given the embolic potential of these masses. Further research is necessary to better understand the pathophysiology of PFEs and to establish standardized management guidelines.

## Conclusions

Although thrombus in the LAA is not uncommon, neoplasms in the LAA are rare, but, when present, they can be significant intracardiac sources of embolism, leading to serious sequelae. A rare LAA mass could be a PFE and can be difficult to distinguish from thrombus/clot. This case series provides valuable insights into the clinical spectrum of PFEs, particularly those attached to the LAA and the PVR. Despite the small sample size, this series represents the largest currently in the literature. Accurate identification and categorization of these masses are challenging, yet critical, to guide appropriate surgical intervention and improve patient outcomes. Therefore, clinicians, cardiologists, and imagers should keep PFE of the LAA/PVR in mind when viewing masses in the LAA.

## Financial Support and Author Disclosure

The authors have reported that they have no relationships relevant to the contents of this paper to disclose.

## Appendix

Video 1 2D Transesophageal Echo Midesophageal Level 2-D TEE midesophageal level, with LAA focus showing the mass attached to the orifice of LAA. 2-D = 2-dimensional; LAA = left atrial appendage; TEE = transesophageal echocardiography

Video 2 2D Transesophageal Echo Midesophageal Level 2-D TEE midesophageal level, with LAA focus showing the small mass attached to the side of the PVR. PVR = pulmonary vein ridge; other abbreviations as in Video 1

Video 3 2D Transesophageal Echo Midesophageal Level 2D TEE midesophageal level, with LAA focus showing the highly mobile mass attached to PVR. Abbreviations as in Videos 1 and 2

Video 4 2D Transesophageal Echo Midesophageal Level 2-D TEE midesophageal level, with LAA focus showing the mass attached to LAA orifice. Abbreviations as in Video 1

Video 5 2D Transesophageal Echo Midesophageal Level 2-D TEE midesophageal level, with LAA focus showing the mass attached to PVR. Abbreviations as in Videos 1 and 2

Video 6 Transesophageal Echo With 3D Reconstruction at Midesophageal Level TEE with 3-D reconstruction at midesophageal level, with LAA focus showing the mobile mass attached to PVR. Abbreviations as in Videos 1 and 2

Video 7 2D Transesophageal Echo Midesophageal Level 2D TEE midesophageal level, with LAA focus showing large mobile mass attached to the PVR. Abbreviations as in Videos 1 and 2

Video 8 2D Transesophageal Echo Midesophageal Level 2-D TEE midesophageal level, with LAA focus showing the small mass attached to PVR. Abbreviations as in Videos 1 and 2

Video 9 2D Transesophageal Echo Midesophageal Level 2-D TEE midesophageal level, with LAA focus showing the mass attached to the orifice of the LAA. Abbreviations as in Video 1
